# Changes in both gene expression and intron retention tune the *Arabidopsis thaliana* heat shock response

**DOI:** 10.17912/micropub.biology.002278

**Published:** 2026-08-12

**Authors:** Yifan Huang, Nick Kaplinsky

**Affiliations:** 1 Department of Biology, Swarthmore College

## Abstract

Plant responses to high temperatures are critical for survival, yet acquired thermotolerance (AT) is limited to a tight temperature range. In Arabidopsis, 34 ^o^ C-37 ^o^ C induces AT while 40 ^o^ C does not. Analyzing RNA-seq data from seedlings acclimated between 37°C-40°C showed that while heat-induced genes are robustly expressed, their functional expression is severely attenuated as temperatures rise due to decreasing expression levels and increasing intron retention. This reduction, particularly for genes with functions in the heat shock response (HSR), suggests that combinatorial changes in expression and splicing play a key role in tuning the plant HSR and limiting AT at higher temperatures.

**Figure 1. Lower mRNA levels and increased intron retention result in decreased expression of heat-induced genes as temperatures increase from 37 o C to 40 o C:** (A) Per-gene expression trajectories are shown for uninduced genes (left) and genes induced at 37 ^o^ C (right) using log _2_ FC >= 1 and p _adj_ < 0.0001 as criteria for induction. For each gene, expression was normalized to its 37 ^o^ C value. Individual genes are shown in blue-grey, *HSA32* and *HSP101* are shown in yellow and orange, and red circles indicate the mean normalized expression of all genes. For clarity, 4000 randomly selected genes (out of 26,009) are shown in the uninduced panel. (B) Mean intron retention in heat-induced, induced HSR, and uninduced genes at 22 ^o^ C, 37 ^o^ C, 38 ^o^ C, 39 ^o^ C, and 40 ^o^ C. Error bars are ± SEM. (C) Unadjusted and IR-adjusted mean expression normalized to 22 ^o^ C levels. Error bars are ± SEM. (D) Unadjusted and IR-adjusted mean expression normalized to 37 ^o^ C levels. For panels C and D, IR transcripts are assumed to be non-functional and thus do not contribute to gene expression. (E) Genome-browser views of RNA-seq coverage for *ACT11* , *HSA32* , and *HSP101* at 22 ^o^ C, 37 ^o^ C, 38 ^o^ C, 39 ^o^ C, and 40 ^o^ C. Colored tracks indicate coverage at each temperature; blue gene models indicate exon–intron structure. All temperatures are reported in ^o^ C.

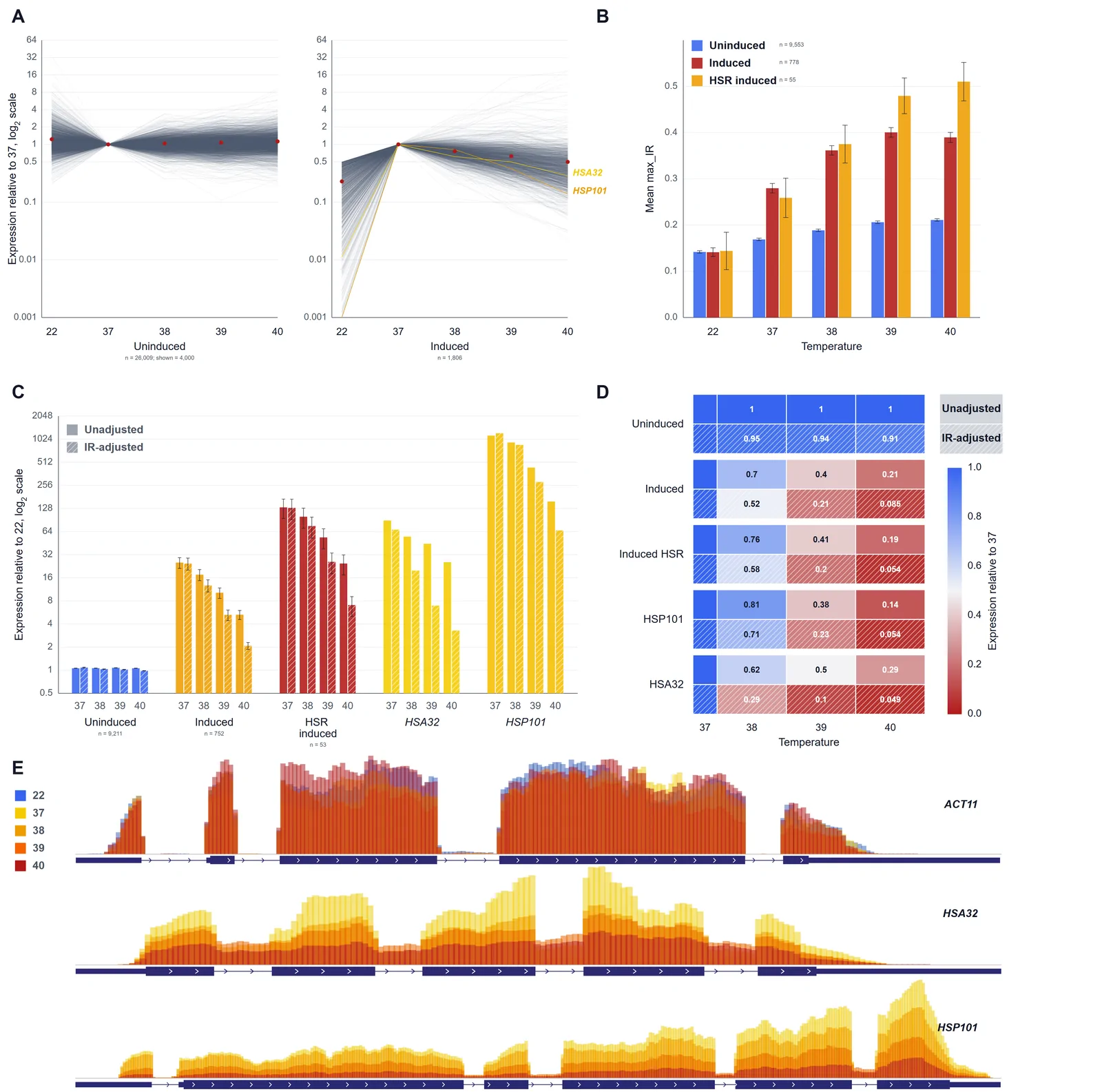



## Description

Plant responses to high temperatures are finely tuned to maximize plant growth and survival. Plants' acquisition of thermotolerance at moderately hot temperatures allows them to survive high temperatures that would otherwise be lethal. Acquired thermotolerance (AT) is mediated by the induction of heat shock response (HSR) genes at acclimation temperatures (Yeh et al. 2012). In Arabidopsis, acclimation at 37 ^o^ C confers AT and protects seedlings from what would otherwise be a lethal 45 ^o^ C heat shock (HS). Acclimation at 40 ^o^ C, a non-lethal HS temperature, does not result in AT (Silva-Correia et al. 2014). To understand the differences in AT between 37°C and 40°C acclimation, we generated RNA-seq data from *Arabidopsis* seedlings incubated at 37°C, 38°C, 39°C, and 40°C for one hour (Gillham et al. 2026).

Compared to plants grown at 22 ^o^ C, plants grown at 37 ^o^ C-40 ^o^ C expressed heat-induced genes enriched for GO terms associated with the HSR such as response to heat and protein folding chaperone. Although GO enrichment did not distinguish between these temperatures, PCA and global sample distance analyses clearly showed degree by degree transcriptional differences. Surprisingly, global patterns of gene expression were more similar at 40 ^o^ C and 22 ^o^ C than at 37 ^o^ C and 22 ^o^ C (Gillham et al. 2026). To investigate the transcriptomic differences that fine-tune the HSR and control whether or not AT occurs, we analyzed gene expression considering intron retention (IR). IR is the most common form of alternative splicing in plants (Petrillo 2023) and occurs at high levels at elevated temperatures (Rosenkranz et al. 2022). Our results suggest that although all temperatures between 37 ^o^ C and 40 ^o^ C result in a robust induction of HSR genes, differences in absolute expression levels coupled with alternative splicing may explain the observed differences in AT at these temperatures.

In order to determine if levels of heat-induced genes differ between 37 ^o^ C-40 ^o^ C samples, we compared the expression of genes induced at 37 ^o^ C vs 22 ^o^ C (heat-induced, log _2_ FC >= 1 and p _adj_ < 0.0001) to uninduced genes. The induced set includes *HSP101* ( At1g74310 ) and *HSA32* ( At4g21320 ), genes required for the acquisition and maintenance of AT, respectively (Charng et al. 2006; Hong and Vierling 2000). The induced genes showed a strong negative temperature trend, with expression decreasing from 37 ^o^ C to 40 ^o^ C. Both *HSP101* and *HSA32* exhibit attenuated expression as temperatures rise from 37 ^o^ C to 40 ^o^ C. In contrast, uninduced genes showed a small but statistically supported increase in expression across the same temperature range ( Fig. 1A ). These trends are statistically supported by both permutation testing and probability analyses (p < 0.00005). Thus, the attenuation of gene expression as temperatures increase is a feature of heat-induced genes rather than a general property of gene expression at high temperatures.

Rates of IR are low at 22 ^o^ C and increase with elevated temperature. IR increased from 37 ^o^ C through 39 ^o^ C, with the highest IR levels observed at 39 ^o^ C for heat-induced genes and a continued modest increase through 40 ^o^ C for uninduced genes. Heat-induced genes showed a much stronger increase in intron retention than uninduced genes, with significantly higher IR at all temperatures from 37 ^o^ C to 40 ^o^ C (p _adj_ <1.46x10 ^-21^ ). In contrast, basal intron retention at 22 ^o^ C was not significantly different between these two groups (p _adj_ =0.13). Analysis of IR in heat-induced genes with annotated HSR functions (including *HSP101* and *HSA32* ) revealed that induced HSR genes exhibit significantly higher IR at 39 ^o^ C (p _adj_ =0.02) and 40 ^o^ C (p _adj_ =0.0018) than heat-induced genes without annotated HSR functions ( Fig. 1B ). These results suggest that there may be specificity in heat-induced IR, although they could also reflect a kinetic model where IR occurs in rapidly transcribed genes associated with accessible chromatin such as the induced HSR genes (Ullah et al. 2018; Zhu et al. 2020; Kodama et al. 2007).

For both *HSP101* and *HSA32* , IR generates transcripts containing premature termination codons that, if translated, would encode truncated and non-functional proteins. Although there are examples of functional IR in Arabidopsis, these appear to be the exception and not the rule and, to the extent they have been characterized, functional IR variants appear to encode dominant negative isoforms (Kim et al. 2016; Liu et al. 2013; Wu et al. 2019).

To estimate the potential effects of IR on the HSR, we made the simplifying assumption that IR transcripts are non-functional and calculated IR-adjusted expression values for each gene as expression x (1 - max_IR). Unadjusted expression patterns of genes for which we have IR data ( Fig. 1C ) are similar to those observed for all genes ( Fig. 1A ). This demonstrates that the subset of genes with complete IR data behaves similarly to larger gene sets that include genes without high-quality IR data. IR increases significantly with temperature for induced and HSR induced genes ( Fig. 1B ), resulting in lower IR-adjusted expression levels compared to unadjusted expression levels for these groups of genes as well as for both *HSP101* and *HSA32* ( Fig. 1C ). Because acclimation at 37 ^o^ C results in strong AT (Silva-Correia et al. 2014) we calculated IR-adjusted expression (functional expression) at higher temperatures normalized to 37 ^o^ C to estimate how the combination of decreased expression and IR might attenuate the HSR ( Fig. 1D ). While the small increase in IR at 40 ^o^ C among uninduced genes results in a ~9% decrease in functional expression, the larger increase in IR combined with significantly decreased expression levels results in a ~91% decrease in functional expression for induced genes. That estimate increases to ~95% for induced HSR genes as a group, as well as for *HSP101* and *HSA32* . Inspection of RNA-seq coverage for an uninduced gene ( *ACT11* At3g12110 ) and for *HSP101* and *HSA32* shows that, while *ACT11* levels and splicing are substantially temperature independent, the combination of reduced expression levels and high levels of IR at 40 ^o^ C observed in *HSP101* and *HSA32* results in few spliced transcripts ( Fig. 1E ).

It seems plausible that the dramatic reduction in the number of functional heat-induced transcripts, especially those with known HSR functions, may explain the lack of AT observed when acclimation is hotter than 37 ^o^ C. This hypothesis could be tested by genetically modulating thermally induced IR or by engineering plants to express intron-free alleles of critical HSR genes.

## Methods

RNA-seq dataset generation

A detailed description of plant growth and treatment conditions, RNA extraction, RNA-seq library construction and sequencing, and the processing and technical validation of the RNA-seq data can be found in Gillham et al. (2026). Briefly, five-day-old Arabidopsis seedlings were exposed to 22 ^o^ C, 37 ^o^ C, 38 ^o^ C, 39 ^o^ C, and 40 ^o^ C for one hour before total RNA was extracted and used to prepare Illumina RNA-seq libraries. RNA-seq reads were trimmed, quality filtered, and mapped to the Arabidopsis genome. Each temperature treatment was replicated in triplicate (Gillham et al. 2026). Read data are available as BioProject ID PRJNA1481274 at the NCBI SRA.

Expression analysis

Differentially expressed genes were identified using DESeq2 v1.40.2 (Love et al. 2014). The heat-induced gene set consisted of 1,806 nuclear genes induced at 37 ^o^ C relative to 22 ^o^ C (log _2_ FC >= 1 and p _adj_ < 0.0001). The comparison set contained 26,009 uninduced genes that had usable DESeq2 data for all 37 ^o^ C to 40 ^o^ C pairwise comparisons to 22 ^o^ C.

Gene-set temperature trends were evaluated using two complementary tests. First, for each gene we calculated the slope of log _2_ -normalized expression across 37 ^o^ C, 38 ^o^ C, 39 ^o^ C, and 40 ^o^ C. We then tested whether the mean slope for each gene set was more negative or more positive than expected by chance using 20,000 within-gene permutations of the temperature labels. When no permutation was as extreme as the observed value, the result is reported as p < 0.00005, the resolution limit of the permutation test.

As a second, order-based test, we counted the number of genes whose expression followed a strictly monotonic decrease across the temperature series: 37 ^o^ C > 38 ^o^ C > 39 ^o^ C > 40 ^o^ C. The probability of this exact ordering is 1/24 if the values are equally likely to occur in any order. We used an exact upper-tail binomial calculation to test whether the observed number of monotonically decreasing genes was greater than expected by chance.

To estimate the abundance of potentially functional transcripts, we assumed that all intron retention events produce non-functional transcripts. We analyzed genes for which we had expression data at all temperatures and IRFinder warning-free data across all samples (see splicing analysis). IR-adjusted expression was calculated as:

(expression _temp_ x (1 - max_IR _temp_ )) / (expression _22_ x (1 - max_IR _22_ )). A small number of genes with max_IR _22_ =1 resulted in undefined IR-adjusted expression values and were excluded from the analysis.

Splicing analysis

Gene-level intron retention was generated using IRFinder v2.0.1 (Lorenzi et al. 2021). For each gene in each sample, the maximum IR ratio (max_IR) observed across all annotated introns was determined. Genes were retained for this analysis only if they had warning-free IR values for all three biological replicates at all temperatures. Three groups of retained genes were used for our IR analysis: 778 heat-induced genes and 9553 uninduced genes (as defined above), and 55 heat-induced genes with annotated HSR functions. The 55 gene heat-induced HSR group was generated by overlapping heat-induced genes, genes with warning-free IR values, and the 263 Arabidopsis genes annotated with cellular response to heat (GO:0034605) or response to heat (GO:0009408) terms. Not all HSR genes are heat-inducible and most sHSPs are intron-free (Scharf et al. 2001). 

For each retained gene, max_IR values from each replicate were averaged and used to calculate group-level max_IR means.

For comparisons involving induced heat-induced HSR genes and heat-induced genes, the heat-induced group was defined as heat-induced genes excluding the induced HSR subset to avoid non-independence caused by comparing a subset of genes directly with the full parent set that contains it. 

Statistical comparisons between temperatures within the same gene set used paired two-sided Wilcoxon signed-rank tests. Comparisons between groups used two-sided Mann-Whitney U tests. P values were corrected across all comparisons using Benjamini-Hochberg correction.
